# Design of a Multiepitope-Based Peptide Vaccine against the E Protein of Human COVID-19: An Immunoinformatics Approach

**DOI:** 10.1155/2020/2683286

**Published:** 2020-05-11

**Authors:** Miyssa I. Abdelmageed, Abdelrahman H. Abdelmoneim, Mujahed I. Mustafa, Nafisa M. Elfadol, Naseem S. Murshed, Shaza W. Shantier, Abdelrafie M. Makhawi

**Affiliations:** ^1^Faculty of Pharmacy, University of Khartoum, Khartoum, Sudan; ^2^Faculty of Medicine, Alneelain University, Khartoum, Sudan; ^3^Department of Biotechnology, University of Bahri, Khartoum, Sudan; ^4^Department of Molecular Biology, National University Biomedical Research Institute, National University, Khartoum, Sudan; ^5^Department of Microbiology, International University of Africa, Khartoum, Sudan; ^6^Department of Pharmaceutical Chemistry, Faculty of Pharmacy, University of Khartoum, Khartoum, Sudan

## Abstract

**Background:**

A new endemic disease has spread across Wuhan City, China, in December 2019. Within few weeks, the World Health Organization (WHO) announced a novel coronavirus designated as coronavirus disease 2019 (COVID-19). In late January 2020, WHO declared the outbreak of a “public-health emergency of international concern” due to the rapid and increasing spread of the disease worldwide. Currently, there is no vaccine or approved treatment for this emerging infection; thus, the objective of this study is to design a multiepitope peptide vaccine against COVID-19 using an immunoinformatics approach.

**Method:**

Several techniques facilitating the combination of the immunoinformatics approach and comparative genomic approach were used in order to determine the potential peptides for designing the T-cell epitope-based peptide vaccine using the envelope protein of 2019-nCoV as a target.

**Results:**

Extensive mutations, insertion, and deletion were discovered with comparative sequencing in the COVID-19 strain. Additionally, ten peptides binding to MHC class I and MHC class II were found to be promising candidates for vaccine design with adequate world population coverage of 88.5% and 99.99%, respectively.

**Conclusion:**

The T-cell epitope-based peptide vaccine was designed for COVID-19 using the envelope protein as an immunogenic target. Nevertheless, the proposed vaccine rapidly needs to be validated clinically in order to ensure its safety and immunogenic profile to help stop this epidemic before it leads to devastating global outbreaks.

## 1. Introduction

Coronaviruses (CoV) are a large family of zoonotic viruses that cause illness ranging from the common cold to more severe diseases such as Middle East Respiratory Syndrome (MERS-CoV) and Severe Acute Respiratory Syndrome (SARS-CoV). In the last decades, six strains of coronaviruses were identified; however, in December 2019, a new strain has spread across Wuhan City, China [1, 2]. It was designated as coronavirus disease 2019 (COVID-19) by the World Health Organization (WHO) [3]. In late January 2020, WHO declared the outbreak a global pandemic with cases in more than 45 countries where the COVID-19 was spreading fast outside China, most significantly in South Korea, Italy, and Iran with over 2,924 deaths and 85,212 cases confirmed while 39,537 recovered on 29 February 2020, 06:05 AM (GMT).

COVID-19 is a positive-sense single-stranded RNA virus (+ssRNA). Its RNA sequence is approximately 30,000 bases in length [4]. It belongs to the subgenus *Sarbecovirus* and genus *Betacoronavirus* within the family *Coronaviridae*. The corona envelope (E) protein is a small, integral membrane protein involved in several aspects of the virus' life cycle, such as pathogenesis, envelope formation, assembly, and budding, alongside with its interactions with both other CoV proteins (M, N, and S) and host cell proteins (release of infectious particles after budding) [5–9].

The infected person is characterized with fever, upper or lower respiratory tract symptoms, diarrhea, lymphopenia, thrombocytopenia, and increased C-reactive protein and lactate dehydrogenase levels or combination of all these within 3-6 days after exposure. Further molecular diagnosis can be made by real-time PCR for genes encoding the internal RNA-dependent RNA polymerase and Spike's receptor binding domain, which can be confirmed by Sanger sequencing and full genome analysis by NGS, multiplex nucleic acid amplification, and microarray-based assays [10–14].

A phylogenetic tree of the mutation history of a family of viruses is possible to reconstruct with a sufficient number of sequenced genomes. The phylogenetic analysis indicates that COVID-19 likely originated from bats [15]. It also showed that it is highly related with at most seven mutations relative to a common ancestor [16].

The sequence of COVID-19 RBD, together with its RBM that contacts receptor angiotensin-converting enzyme 2 (ACE2), was found similar to that of SARS coronavirus. In January 2020, a group of scientists demonstrated that ACE2 could act as the receptor for COVID-19 [17–21]. However, COVID-19 differs from other previous strains in having several critical residues at the 2019-nCoV receptor-binding motif (particularly Gln493) which provide advantageous interactions with human ACE2 [15]. This difference in affinity possibly explains why the novel coronavirus is more contagious than other viruses.

At present, there is no vaccine or approved treatment for humans, but Chinese traditional medicines, such as ShuFengJieDu capsules and Lianhuaqingwen capsules, could be possible treatments for COVID-19. However, there are no clinical trials approving the safety and efficacy for these drugs [22].

The main concept within all the immunizations is the ability of the vaccine to initiate an immune response in a faster mode than the pathogen itself. Although traditional vaccines, which depend on biochemical trials, induced potent neutralizing and protective responses in the immunized animals, they can be costly, allergenic, and time-consuming and require in vitro culture of pathogenic viruses leading to serious concern of safety [23, 24]. Thus, the need for safe and efficacious vaccines is highly recommended.

Peptide-based vaccines do not need in vitro culture making them biologically safe, and their selectivity allows accurate activation of immune responses [25, 26]. The core mechanism of the peptide vaccines is built on the chemical method to synthesize the recognized B-cell and T-cell epitopes that are immunodominant and can induce specific immune responses. A B-cell epitope of a target molecule can be linked with a T-cell epitope to make it immunogenic. The T-cell epitopes are short peptide fragments (8-20 amino acids), whereas the B-cell epitopes can be proteins [27, 28]. Therefore, in this study, we aimed to design a peptide-based vaccine to predict epitopes from the corona envelope (E) protein using immunoinformatics analysis [29–34]. Rapid further studies are recommended to prove the efficiency of the predicted epitopes as a peptide vaccine against this emerging infection.

## 2. Materials and Methods

**Figure 1:**
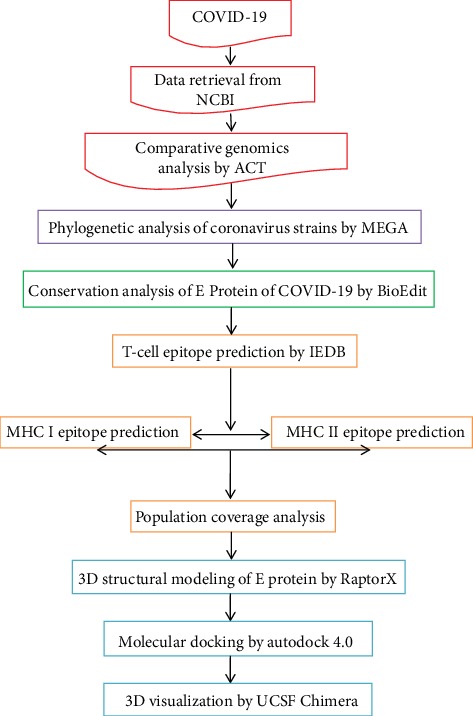
Descriptive workflow for the epitope-based peptide vaccine prediction.

The workflow summarizing the procedures for the epitope-based peptide vaccine prediction is shown in Figure 1.

### 2.1. Data Retrieval

Full GenBank files of the complete genomes and annotation of COVID-19 (NC_04551), SARS-CoV (FJ211859), MESA-CoV (NC_019843), HCoV-HKU1 (AY884001), HCoV-OC43 (KF923903), HCoV-NL63 (NC_005831), and HCoV-229E (KY983587) were retrieved from the National Center for Biotechnology Information (NCBI), while the FASTA format of the envelope (E) protein (YP_009724392.1), spike (S) protein (YP_009724390.1), nucleocapsid (N) protein (YP_009724397.2), and membrane (M) protein (YP_009724393.1) of 2019-nCoV and the envelope (E) protein of two Chinese and two American sequences (YP009724392.1, QHQ71975.1, QHO60596.1, and QHN73797.1) were obtained from the NCBI (https://www.ncbi.nlm.nih.gov/).

### 2.2. The Artemis Comparison Tool (ACT)

ACT is an in silico analysis software for visualization of comparisons between complete genome sequences and associated annotations [35]. It is also applied to identify regions of similarity, rearrangements, and insertions at any level from base pair differences to the whole genome (https://www.sanger.ac.uk/science/tools/artemis-comparison-tool-act).

### 2.3. VaxiJen Server

It is the first server for alignment-independent prediction of protective antigens. It allows antigen classification solely based on the physicochemical properties of proteins without recourse to sequence alignment. It predicts the probability of the antigenicity of one or multiple proteins based on auto cross covariance (ACC) transformation of protein sequence. Structural CoV-2019 proteins (N, S, E, and M) were analyzed by VaxiJen with threshold of 0.4 [36] (http://www.ddg-pharmfac.net/vaxijen/VaxiJen/VaxiJen.html).

### 2.4. BioEdit

It is a software package proposed to stream a distinct program that can run nearly any sequence operation as well as a few basic alignment investigations. The sequences of the E protein retrieved from UniProt were run in BioEdit to determine the conserved sites through ClustalW in the application settings [37].

### 2.5. The Molecular Evolutionary Genetics Analysis (MEGA)

MEGA (version 10.1.6) is software for the comparative analysis of molecular sequences. It is used for pairwise and multiple sequence alignment alongside construction and analysis of phylogenetic trees and evolutionary relationships. The gap penalty was 15 for opening and 6.66 for extending the gap for both pairwise and multiple sequence alignment. Bootstrapping of 300 was used in construction of the maximum like hood phylogenetic tree [38, 39] (https://www.megasoftware.net).

### 2.6. Prediction of T-Cell Epitopes

IEDB tools were used to predict the conserved sequences (10-mer sequence) from HLA class I and class II T-cell epitopes by using an Artificial Neural Network (ANN) approach [40–42]. The Artificial Neural Network (ANN) version 2.2 was chosen as the prediction method as it depends on the median inhibitory concentration (IC50) [40, 43–45]. For the binding analysis, all the alleles were carefully chosen, and the length was set at 10 before prediction was done. Analysis of epitopes binding to the MHC class I and II molecules was assessed by the IEDB MHC prediction server at http://tools.iedb.org/mhci/ and http://tools.iedb.org/mhcii/, respectively. All conserved immunodominant peptides binding to the MHC I and II molecules at scores equal or less than 100 median inhibitory concentrations (IC50) and 1000, respectively, were selected for further analysis while epitopes with IC50 greater than 100 were eliminated [46].

### 2.7. Population Coverage Analysis

Population coverage for each epitope was carefully determined by the IEDB population coverage calculation tool. Due to the diverse binding sites of epitopes with different HLA alleles, the most promising epitope candidates were calculated for population coverage against the population of the whole world, China, and Europe to get and ensure a universal vaccine [47, 48] (http://tools.iedb.org/population/).

### 2.8. Tertiary Structure (3D) Modeling

The reference sequence of the E protein that has been retrieved from GenBank was used as an input in RaptorX to predict the 3D structure of the E protein [49, 50]; the visualization of the obtained 3D protein structure was performed in UCSF Chimera (version1.8) [51].

### 2.9. In Silico Molecular Docking

#### 2.9.1. Ligand Preparation

In order to estimate the binding affinities between the epitopes and the molecular structure of MHC I and MHC II, in silico molecular docking was used. Sequences of proposed epitopes were selected from the COVID-19 reference sequence using UCSF Chimera 1.10 and saved as a PDB file. The obtained files were then optimized and energy minimized. The HLA-A∗02:01 was selected as the macromolecule for docking. Its crystal structure (4UQ3) was downloaded from the RCSB Protein Data Bank (http://www.rcsb.org/pdb/home/home.do), which was in a complex with an azobenzene-containing peptide [52].

All water molecules and heteroatoms in the retrieved target file 4UQ3 were then removed. The target structure was further optimized and energy minimized using Swiss PDB Viewer V.4.1.0 software [53].

#### 2.9.2. Molecular Docking

Molecular docking was performed using AutoDock 4.0 software, based on the Lamarckian genetic algorithm, which combines energy evaluation through grids of affinity potential to find the suitable binding position for a ligand on a given protein [54, 55] Polar hydrogen atoms were added to the protein targets, and Kollman united atomic charges were computed. The target's grid map was calculated and set to 60 × 60 × 60 points with grid spacing of 0.375 Ǻ. The grid box was then allocated properly in the target to include the active residue in the center. The genetic algorithm and its run were set to 100. The docking algorithms were set to default. Finally, results were retrieved as binding energies and poses that showed the lowest binding energies visualized using UCSF Chimera.

## 3. Results

### 3.1. The Artemis Comparison Tool

**Figure 2:**
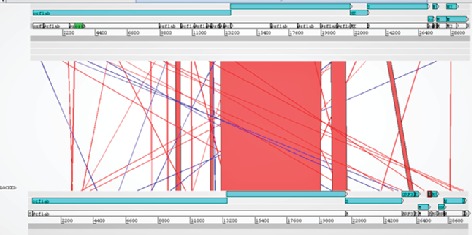
Artemis analysis of the envelope protein displaying 3 windows. The upper window represents the HCoV-HKU1 reference sequence, and its genes are highlighted in blue starting from *orflab* gene and ending with *N* gene. The middle window describes the similarities and the difference between the two genomes. Red lines indicate a match between genes from the two genomes; blue lines indicate inversion which represents the same sequences in the two genomes, but they are organized in the opposite direction. The lower window represents COVID-19 and its genes starting from *orflab* gene and ending with *N* gene.

The reference sequence of the envelope protein was aligned with the HCoV-HKU1 reference protein using the Artemis Comparison Tool as illustrated in (Figure 2).

### 3.2. VaxiJen Server

The mutated proteins were tested for antigenicity using VaxiJen software, where the envelope protein was found as the best immunogenic target in Table 1.

### 3.3. BioEdit

**Figure 3:**
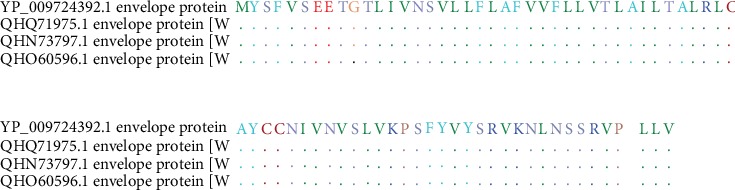
Sequence alignment of the envelope protein of COVID-19 using BioEdit software (total conservation through the 4 strains: 2 from China and 2 from the USA).

Sequence alignment of the COVID-19 envelope protein was done using BioEdit software which shows total conservation across four sequences which were retrieved from China and the USA (Figure 3).

### 3.4. The Molecular Evolutionary Genetics Analysis

**Figure 4:**
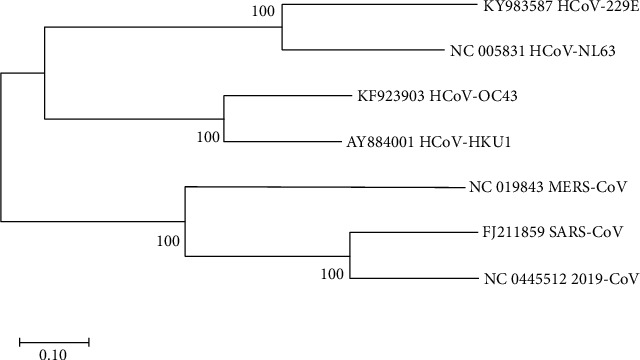
Maximum likelihood phylogenetic tree which describes the evolutionary relationship between the seven strains of coronavirus.

To study the evolutionary relationship between all the seven strains of coronavirus, a multiple sequence alignment (MSA) was performed using ClustalW by MEGA software. This alignment was used to construct the maximum likelihood phylogenetic tree as seen in Figure 4.

### 3.5. Prediction of T-Cell Epitopes and Population Coverage

**Figure 5:**
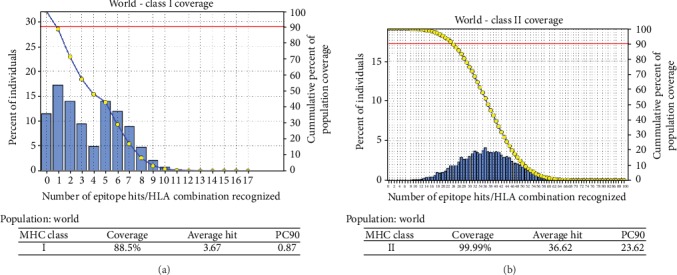
Schematic diagrams (a) and (b) showing world population coverage of the envelope protein of COVID-19 binding to the MHC class I and MHC class II molecules, respectively.

**Figure 6:**
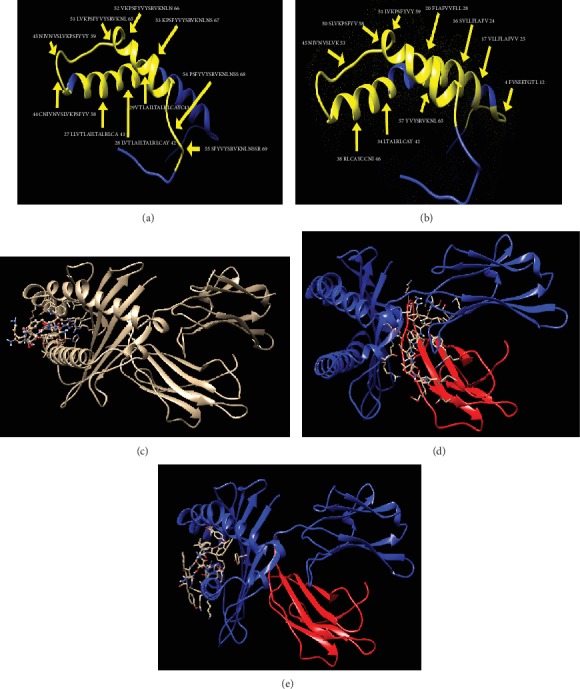
3D structures visualized by UCSF Chimera: (a) and (b) show the most promising peptides in the envelope protein of COVID-19 (yellow colored) binding to MHC class I and MHC class II, respectively, while (c), (d), and (e) show the molecular docking of the YVYSRVKNL, LAILTALRL, and SLVKPSFYV peptides of coronavirus docked in HLA-A∗02:01, respectively.

The IEDB website was used to analyze the 2019-nCoV envelope protein for T-cell-related peptides. Results show ten MHC class I- and II-associated peptides with high population coverage (Tables 2 and 3; Figure 5). The most promising peptides were visualized using UCSF Chimera software (Figures 6(a) and 6(b)).

## 4. Discussion

Designing a novel vaccine is very crucial to defend against the rapid endless global burden of diseases [56–59]. In the last few decades, biotechnology has advanced rapidly, alongside with the understanding of immunology which assisted the rise of new approaches towards rational vaccine design [60]. Peptide-based vaccines are designed to elicit immunity particular pathogens by selectively stimulating antigen-specific B- and T-cells [25]. Applying the advanced bioinformatics tools and databases, various peptide-based vaccines could be designed where the peptides act as ligands [61–63]. This approach has been used frequently in Saint Louis encephalitis virus [64], dengue virus [65], and Chikungunya virus [66] proposing promising peptides for designing vaccines.

The COVID-19 is an RNA virus which tends to mutate more commonly than the DNA viruses [67]. These mutations lie on the surface of the protein, which makes COVID-19 more superior than other previous strains by inducing its sustainability leaving the immune system in a blind spot [68].

In our present work, different peptides were proposed for designing a vaccine against COVID-19 (Figure 1). In the beginning, the whole genome of COVID-19 was analyzed by a comparative genomic approach to determine the potential antigenic target [69]. The Artemis Comparison Tool (ACT) was used to analyze human coronavirus (HCoV-HKU1) reference sequence vs. Wuhan-Hu-1 COVID-19. Results obtained (Figure 2) revealed extensive mutations among the tested genomes. New genes (*ORF8* and *ORF6*) were found inserted in COVID-19 which were absent in HCoV-HKU1 that might be acquired by the horizontal gene transmission [70]. The high rate of mutation between the two genomes was observed in the region from 20,000 bp to the end of the sequence. This region encodes the four major structural proteins in coronavirus which are the envelope (E) protein, nucleocapsid (N) protein, membrane (M) protein, and spike (S) protein, all of which are required to produce a structurally complete virus [71, 72].

These conserved antigenic sites were revealed in previous studies through sequence alignment between MERS-CoV and bat coronavirus [73] and analyzed in SARS-CoV [74].

The four proteins were then analyzed by VaxiJen software to test the probability of antigenic proteins. Protein E was found to be the most antigenic gene with the highest probability as shown in Table 1. A literature survey confirmed this result in which protein E was investigated in Severe Acute Respiratory Syndrome (SARS) in 2003 and, more recently, Middle-East Respiratory Syndrome (MERS) [71]. Furthermore, the conservation of this protein against the seven strains was tested and confirmed through the use of the BioEdit package tool (Figure 3).

Phylogenetic analysis is a very powerful tool for determining the evolutionary relationship between strains. Multiple sequence alignment (MSA) was performed using ClustalW for the seven strains of coronavirus, which are COVID-19 (NC_04551), SARS-CoV (FJ211859), MESA-CoV (NC_019843), HCoV-HKU1 (AY884001), HCoV-OC43 (KF923903), HCoV-NL63 (NC_005831), and HCoV-229E (KY983587). The maximum likelihood phylogenetic tree revealed that COVID-19 is found in the same clade of SARS-CoV; thus, the two strains are highly related to each other (Figure 4).

The immune response of T-cells is considered a long-lasting response compared to B-cells, where the antigen can easily escape the antibody memory response [75]. Vaccines that effectively generate cell-mediated responses are needed to provide protection against the invading pathogen. Moreover, the CD8+ and CD4+ T-cell responses play a major role in antiviral immunity [76]. Thus, designing a vaccine against T-cells is much more important.

Choosing protein E as the antigenic site, the binding affinity to MHC molecules was then evaluated. The protein reference sequence was submitted to the IEDB MHC predication tool. 21 peptides were found to bind MHC class I with different affinities (Table 1), from which ten peptides were selected for vaccine design based on the number of alleles and world population percentage (Table 2; Figure 5). Analysis in the IEDB MHC II binding prediction tool resulted in prediction of 61 peptides (Table 2), from which ten peptides were selected for vaccine design based on the number of alleles and world population percentage (Table 3; Figure 5). Unfortunately, IEDB did not give any result for B-cell epitopes; this might be due to the length of the COVID-19 (75 amino acids).

It is well known that peptides recognized with a high number of HLA molecules are potentially inducing immune response. Based on the aforementioned results and taking into consideration the high binding affinity to both MHC class I and II, conservancy, and population coverage, three peptides are strongly proposed to formulate a new vaccine against COVID-19.

These findings were further confirmed by the results obtained for the molecular docking of the proposed peptides and HLA-A∗02:01. The formed complex between the MHC molecule and the three peptides (YVYSRVKNL, SLVKPSFYV, and LAILTALRL) has shown peptide amino- and carboxyl-termini forming one and three hydrogen bonds, respectively, at the two ends of a binding groove with MHC residues with the least binding energy -13.2 kcal/mol, -11 kcal/mol, and -11.3 kcal/mol, respectively (Figures 6(c)–6(e)).

Although both flu and anti-HIV drugs are used currently in China for treatment of COVID-19, chloroquine phosphate, an old drug for treatment of malaria, has recently been found to have apparent efficacy and acceptable safety against COVID-19 [77, 78]; nevertheless, more studies are required to standardize these therapies. In addition, there has been some success in the development of mouse models of MERS-CoV and SARS-CoV infection, and candidate vaccines where the envelope (E) protein is mutated or deleted have been described [79–85]. To the best of our knowledge, this is the first study to identify certain peptides in the envelope (E) protein as candidates for COVID-19. Accordingly, these epitopes were strongly recommended as promising epitope vaccine candidates against T-cells.

## 5. Conclusion

Extensive mutations, insertion, and deletion were discovered in the COVID-19 strain using the comparative sequencing. In addition, a number of the MHC class I- and II-related peptides were found to be promising candidates. Among which, the peptides YVYSRVKNL, SLVKPSFYV, and LAILTALRL show high potentiality for vaccine design with adequate world population coverage. The T-cell epitope-based peptide vaccine was designed for COVID-19 using the envelope protein as an immunogenic target; nevertheless, the proposed vaccine rapidly needs to be validated clinically ensuring its safety and immunogenic profile to help stop this epidemic before it leads to devastating global outbreaks.

## Figures and Tables

**Table 1:** VaxiJen overall prediction of probable COVID-19 antigen.

Protein	Result	VaxiJen prediction
Protein E	0.6025	Probable antigen
Protein M	0.5102	Probable antigen
Protein S	0.4646	Probable antigen
Protein N	0.5059	Probable antigen

**Table 2:** The most promising MHC class I-related peptides in the envelope protein-based vaccine of COVID-19 along with the predicted coverage of the world, China, Europe, and East Asia.

Peptide	Alleles	Coverage	Combined coverage of 10 peptides
YVYSRVKNL	HLA-C∗14:02, HLA-C∗12:03, HLA-C∗07:01, HLA-C∗03:03, HLA-C∗06:02	50.02%	World: 88.5%
SLVKPSFYV	HLA-A∗02:06, HLA-A∗02:01, HLA-A∗68:02	42.53%	China: 78.17%
SVLLFLAFV	HLA-A∗02:06, HLA-A∗68:02, HLA-A∗02:01	42.53%	Europe: 92.94%
FLAFVVFLL	HLA-A∗02:01, HLA-A∗02:06	40.60%	East Asia: 80.78%
VLLFLAFVV	HLA-A∗02:01	39.08%	
RLCAYCCNI	HLA-A∗02:01	39.08%	
FVSEETGTL	HLA-C∗03:03, HLA-C∗12:03, HLA-A∗02:06, HLA-A∗68:02, HLA-B∗35:01	28.22%	
LTALRLCAY	HLA-A∗01:01, HLA-A∗30:02, HLA-B∗15:01	26.34%	
LVKPSFYVY	HLA-B∗15:01, HLA-A∗29:02, HLA-A∗30:02, HLA-B∗35:01	21.72%	
NIVNVSLVK	HLA-A∗68:01, HLA-A∗11:01	20.88%	

**Table 3:** The most promising MHC class II-related peptides in the envelope protein-based vaccine of COVID-19 along with the predicted coverage of the world, China, Europe, and East Asia.

Peptide sequence	Alleles	World coverage	Coverage/10 peptides
KPSFYVYSRVKNLNS	HLA-DPA1∗01:03, HLA-DPB1∗02:01, HLA-DPB1∗03:01, HLA-DPB1∗04:01, HLA-DPA1∗02:01, HLA-DPB1∗05:01, HLA-DPA1∗03:01, HLA-DPB1∗04:02, HLA-DPB1∗06:01, HLA-DPB1∗14:01, HLA-DPB1∗01:01, HLA-DQA1∗05:01, HLA-DQB1∗04:02, HLA-DQA1∗06:01, HLA-DQA1∗01:02, HLA-DQB1∗05:01, HLA-DQA1∗02:01, HLA-DRB1∗01:01, HLA-DRB1∗07:01, HLA-DRB1∗08:01, HLA-DRB1∗09:01, HLA-DRB1∗11:01, HLA-DRB4∗01:03, HLA-DRB1∗04:01, HLA-DRB1∗10:01, HLA-DRB1∗04:05, HLA-DRB1∗13:01, HLA-DRB1∗08:02, HLA-DRB1∗16:02, HLA-DRB1∗15:01, HLA-DRB3∗03:01, HLA-DRB5∗01:01, HLA-DRB3∗02:02, HLA-DRB1∗04:04, HLA-DRB1∗13:02	99.93%	World: 99.99%
VKPSFYVYSRVKNLN	HLA-DPA1∗01:03, HLA-DPB1∗02:01, HLA-DPB1∗04:01, HLA-DPB1∗03:01, HLA-DPA1∗02:01, HLA-DPB1∗05:01, HLA-DPA1∗03:01, HLA-DPB1∗04:02, HLA-DPB1∗06:01, HLA-DPB1∗01:01, HLA-DQA1∗05:01, HLA-DQB1∗04:02, HLA-DQA1∗06:01, HLA-DQA1∗01:02, HLA-DQB1∗05:01, HLA-DQA1∗02:01, HLA-DRB1∗07:01, HLA-DRB1∗08:01, HLA-DRB1∗01:01, HLA-DRB1∗09:01, HLA-DRB1∗11:01, HLA-DRB4∗01:03, HLA-DRB1∗15:01, HLA-DRB1∗13:01, HLA-DRB3∗03:01, HLA-DRB1∗10:01, HLA-DRB1∗16:02, HLA-DRB1∗08:02, HLA-DRB1∗04:05, HLA-DRB5∗01:01, HLA-DRB1∗13:02, HLA-DRB3∗02:02, HLA-DRB1∗04:01, HLA-DRB1∗04:04	99.92%	China: 99.96%
LVKPSFYVYSRVKNL	HLA-DPA1∗01:03, HLA-DPB1∗02:01, HLA-DPB1∗04:01, HLA-DPA1∗02:01, HLA-DPB1∗05:01, HLA-DPB1∗06:01, HLA-DPA1∗03:01, HLA-DPB1∗04:02, HLA-DPA1∗02:01, HLA-DPB1∗01:01, HLA-DQA1∗06:01, HLA-DQB1∗04:02, HLA-DQA1∗05:01, HLA-DQA1∗02:01, HLA-DQA1∗01:04, HLA-DQB1∗05:03, HLA-DQA1∗01:02, HLA-DQB1∗05:01, HLA-DRB1∗07:01, HLA-DRB1∗08:01, HLA-DRB1∗09:01, HLA-DRB1∗11:01, HLA-DRB4∗01:03, HLA-DRB3∗03:01, HLA-DRB1∗01:01, HLA-DRB1∗15:01, HLA-DRB1∗16:02, HLA-DRB1∗13:01, HLA-DRB1∗10:01, HLA-DRB1∗08:02, HLA-DRB5∗01:01, HLA-DRB1∗13:02, HLA-DRB1∗04:05, HLA-DRB3∗02:02, HLA-DRB1∗04:01	99.90%	Europe: 100.0%
PSFYVYSRVKNLNSS	HLA-DPA1∗01:03, HLA-DPB1∗02:01, HLA-DPB1∗03:01, HLA-DPB1∗04:01, HLA-DPA1∗03:01, HLA-DPB1∗04:02, HLA-DPA1∗02:01, HLA-DPB1∗05:01, HLA-DPB1∗06:01, HLA-DQA1∗05:01, HLA-DQB1∗04:02, HLA-DQA1∗01:02, HLA-DQB1∗05:01, HLA-DQA1∗06:01, HLA-DRB1∗01:01, HLA-DRB1∗08:01, HLA-DRB1∗04:01, HLA-DRB1∗11:01, HLA-DRB1∗09:01, HLA-DRB1∗07:01, HLA-DRB4∗01:03, HLA-DRB1∗04:05, HLA-DRB1∗10:01, HLA-DRB1∗13:01, HLA-DRB1∗08:02, HLA-DRB1∗16:02, HLA-DRB1∗15:01, HLA-DRB3∗03:01, HLA-DRB3∗02:02, HLA-DRB1∗04:04, HLA-DRB5∗01:01, HLA-DRB1∗13:02	99.86%	East Asia:99.91%
NIVNVSLVKPSFYVY	HLA-DPA1∗01:03, HLA-DPB1∗02:01, HLA-DPB1∗04:01, HLA-DPB1∗06:01, HLA-DPA1∗02:01, HLA-DPB1∗01:01, HLA-DQA1∗01:02, HLA-DQB1∗05:01, HLA-DQA1∗05:01, HLA-DQB1∗04:02, HLA-DQA1∗02:01, HLA-DQB1∗03:01, HLA-DQB1∗03:03, HLA-DQB1∗03:03, HLA-DQB1∗04:02, HLA-DRB1∗12:01, HLA-DRB1∗01:01, HLA-DRB5∗01:01, HLA-DRB3∗03:01, HLA-DRB1∗13:01, HLA-DRB1∗07:01, HLA-DRB1∗15:01, HLA-DRB4∗01:03, HLA-DRB1∗04:04, HLA-DRB1∗08:02, HLA-DRB1∗09:01, HLA-DRB1∗13:02, HLA-DRB1∗11:01, HLA-DRB1∗04:05, HLA-DRB1∗10:01	99.77%	
LLVTLAILTALRLCA	HLA-DPA1∗01:03, HLA-DPB1∗02:01, HLA-DPB1∗06:01, HLA-DPA1∗03:01, HLA-DPB1∗04:02, HLA-DQA1∗01:02, HLA-DQB1∗05:01, HLA-DQA1∗02:01, HLA-DQB1∗03:01, HLA-DQB1∗03:03, HLA-DQA1∗05:01, HLA-DQB1∗04:02, HLA-DQA1∗06:01, HLA-DQA1∗01:03, HLA-DQB1∗06:03, HLA-DRB4∗01:03, HLA-DRB1∗01:01, HLA-DRB1∗13:01, HLA-DRB1∗04:04, HLA-DRB5∗01:01, HLA-DRB3∗03:01, HLA-DRB1∗10:01, HLA-DRB1∗15:01, HLA-DRB1∗07:01, HLA-DRB1∗11:01, HLA-DRB1∗08:01, HLA-DRB1∗12:01, HLA-DRB1∗03:01, HLA-DRB4∗01:01, HLA-DRB1∗16:02, HLA-DRB1∗08:02	99.72%	
SFYVYSRVKNLNSSR	HLA-DPA1∗01:03, HLA-DPB1∗03:01, HLA-DPB1∗02:01, HLA-DPA1∗03:01, HLA-DPB1∗04:02, HLA-DPA1∗02:01, HLA-DPB1∗05:01, HLA-DPB1∗06:01, HLA-DQA1∗05:01, HLA-DQB1∗04:02, HLA-DQA1∗01:02, HLA-DQB1∗05:01, HLA-DRB1∗04:01, HLA-DRB1∗01:01, HLA-DRB1∗11:01, HLA-DRB1∗08:01, HLA-DRB1∗07:01, HLA-DRB1∗09:01, HLA-DRB4∗01:03, HLA-DRB1∗10:01, HLA-DRB1∗13:01, HLA-DRB1∗04:05, HLA-DRB1∗08:02, HLA-DRB1∗16:02, HLA-DRB3∗02:02, HLA-DRB3∗03:01, HLA-DRB1∗04:04, HLA-DRB1∗15:01, HLA-DRB5∗01:01, HLA-DRB1∗13:02	99.72%	
LVTLAILTALRLCAY	HLA-DPA1∗01:03, HLA-DPB1∗02:01, HLA-DPB1∗06:01, HLA-DPA1∗03:01, HLA-DPB1∗04:02, HLA-DQA1∗01:02, HLA-DQB1∗05:01, HLA-DQA1∗02:01, HLA-DQB1∗03:01, HLA-DQB1∗03:03, HLA-DQA1∗05:01, HLA-DQB1∗04:02, HLA-DQB1∗06:02, HLA-DQA1∗02:01, HLA-DQA1∗06:01, HLA-DRB4∗01:03, HLA-DRB1∗01:01, HLA-DRB1∗13:01, HLA-DRB1∗04:04, HLA-DRB1∗12:01, HLA-DRB1∗10:01, HLA-DRB5∗01:01, HLA-DRB1∗15:01, HLA-DRB1∗11:01, HLA-DRB3∗03:01, HLA-DRB1∗03:01, HLA-DRB1∗08:01, HLA-DRB1∗07:01, HLA-DRB4∗01:01, HLA-DRB1∗16:02, HLA-DRB1∗04:02, HLA-DRB1∗08:02	99.69%	
VTLAILTALRLCAYC	HLA-DPA1∗01:03, HLA-DPB1∗06:01, HLA-DPA1∗03:01, HLA-DPB1∗04:02, HLA-DQA1∗02:01, HLA-DQB1∗03:01, HLA-DQA1∗01:02, HLA-DQB1∗05:01, HLA-DQB1∗06:02, HLA-DQA1∗05:01, HLA-DQB1∗04:02, HLA-DQB1∗03:03, HLA-DQA1∗06:01, HLA-DQB1∗04:02, HLA-DRB1∗01:01, HLA-DRB4∗01:03, HLA-DRB1∗13:01, HLA-DRB1∗04:04, HLA-DRB1∗12:01, HLA-DRB1∗10:01, HLA-DRB5∗01:01, HLA-DRB1∗15:01, HLA-DRB1∗11:01, HLA-DRB1∗03:01, HLA-DRB3∗03:01, HLA-DRB1∗08:01, HLA-DRB1∗07:01, HLA-DRB4∗01:01, HLA-DRB1∗04:02	99.56%	
CNIVNVSLVKPSFYV	HLA-DPA1∗01:03, HLA-DPB1∗06:01, HLA-DPB1∗04:02, HLA-DQA1∗01:02, HLA-DQB1∗05:01, HLA-DQA1∗05:01, HLA-DQB1∗04:02, HLA-DQA1∗02:01, HLA-DQB1∗03:01, HLA-DQB1∗03:03, HLA-DQA1∗01:03, HLA-DQB1∗06:03, HLA-DRB3∗03:01, HLA-DRB1∗12:01, HLA-DRB5∗01:01, HLA-DRB1∗01:01, HLA-DRB1∗07:01, HLA-DRB4∗01:03, HLA-DRB1∗13:01, HLA-DRB1∗15:01, HLA-DRB1∗08:02, HLA-DRB1∗04:04, HLA-DRB1∗09:01, HLA-DRB1∗13:02, HLA-DRB1∗11:01, HLA-DRB1∗04:05, HLA-DRB4∗01:01, HLA-DRB1∗10:01	99.53%	

## Data Availability

All data underlying the results are available as part of the article, and no additional source data are required.

## Abbreviations

WHO: World Health Organization
CoV: Coronaviruses
MERS-CoV: Middle East Respiratory Syndrome
SARS-CoV: Severe Acute Respiratory Syndrome
COVID-19: Novel coronavirus
HCoV-HKU1: Human coronavirus HKU1
HCoV-OC43: Human coronavirus OC43
HCoV-NL63: Human coronavirus NL63
HCoV-229E: Human coronavirus 229E
ACE2: Angiotensin-converting enzyme 2
RBM: Receptor-binding motif
RBD: Receptor-binding domain
vs.: Versus
ACT: Artemis Comparison Tool
ACC: Auto cross covariance
MEGA: Molecular Evolutionary Genetics Analysis
ANN: Artificial Neural Network
IEDB: Immune Epitope Database
IC50: Median inhibitory concentrations
MHC I: Major histocompatibility complex class I
MHC II: Major histocompatibility complex class II
PDB: Protein database
MSA: Multiple sequence alignment.
